# Effects of Electroacupuncture on Pain Threshold of Laboring Rats and the Expression of Norepinephrine Transporter and *α*2 Adrenergic Receptor in the Central Nervous System

**DOI:** 10.1155/2016/9068257

**Published:** 2016-07-28

**Authors:** Qianli Tang, Qiuyan Jiang, Suren R. Sooranna, Shike Lin, Yuanyuan Feng, Qi Zhang, Meili Wang, Yu Wang

**Affiliations:** ^1^Youjiang Medical University for Nationalities, Key Lab of Western Guangxi High Incident Disease, Baise, Guangxi 533000, China; ^2^Department of Gynaecology and Obstetrics, The First Affiliated Hospital of Guangxi University of Chinese Medicine, Nanning, Guangxi 530023, China; ^3^Chelsea & Westminster Hospital, Imperial College London, London SW10 9NH, UK

## Abstract

To observe the effects of electroacupuncture on pain threshold of laboring rats and the expression of norepinephrine transporter and *α*2 adrenergic receptor in the central nervous system to determine the mechanism of the analgesic effect of labor. 120 pregnant rats were divided into 6 groups: a control group, 4 electroacupuncture groups, and a meperidine group. After interventions, the warm water tail-flick test was used to observe pain threshold. NE levels in serum, NET, and *α*2AR mRNA and protein expression levels in the central nervous system were measured. No difference in pain threshold was observed between the 6 groups before intervention. After intervention, increased pain thresholds were observed in all groups except the control group with a higher threshold seen in the electroacupuncture groups. Serum NE levels decreased in the electroacupuncture and MP groups. Increases in NET and *α*2AR expression in the cerebral cortex and decreases in enlarged segments of the spinal cord were seen. Acupuncture increases uptake of NE via cerebral NET and decreases its uptake by spinal NET. The levels of *α*2AR are also increased and decreased, respectively, in both tissues. This results in a decrease in systemic NE levels and may be the mechanism for its analgesic effects.

## 1. Introduction

According to the pain index, labor pain is just secondary only to burning pain. To relieve the laboring pain, common methods used at present, such as epidural puncture, may present the mother with certain risks and medication analgesia as well as produce side effects on the infant by introducing medicine into fetus through placenta. Nowadays there has been a focus on research on analgesia by noninvasive and drug-free methods. Electroacupuncture is a form of acupuncture where a small electric current is passed between pairs of acupuncture needles and this is generally thought to be particularly good for treating different types of pain. Our research group has found that electroacupuncture could relive labor pain [1]. Presently research suggests that norepinephrine (NE) binds to the alpha 2 adrenergic receptor (*α*2AR) to induce analgesia and the norepinephrine transporter (NET) could reabsorb the NE released from the neuron back to the presynaptic membrane as a way to regulate NE levels. By using real-time PCR and western blot analysis, the research presented here attempts to unravel the mechanism of acupuncture analgesia. This is done by exploring the effects of electroacupuncture on NET and *α*2AR mRNA and protein expression in the central nervous system of laboring rats.

## 2. Materials and Methods

### 2.1. Animals

Healthy adult specific-pathogen-free Sprague-Dawley rats, 150 females and 50 males, 3-month-old, sexually mature, weighing 300 g ± 50 g, were provided by the Experimental Animal Center of Guangxi Medical University (Qualification number SCXK Guangxi 2009-0002). Rats were fed under conditions free of specific pathogens at 22–25°C and kept in an environment of 40–60% relative humidity in the Animal Research Institute of Guangxi University of Chinese Medicine. All procedures involving rats were approved by the Committee on the Ethics of Animal Experiments of Guangxi University of Chinese Medicine and were carried out in accordance with the National Institute of Health guidelines. Females and males at a ratio of 1 : 1 were reared in cages, and daily morning checks were conducted. If a vaginal plug was identified, rats were moved out and labeled for observation until abdominal expansion confirmed a pregnancy. From the above cohort, 120 pregnant rats were divided randomly into a control group, Sanyinjiao (SP 6) group, Hegu (LI 4) group, Hegu (LI 4) and Sanyinjiao (SP 6) group, Xuehai (SP 10) group, and a medication group, with 20 in each group.

### 2.2. Treatment and Intervention

(1) There was no intervention for control group, and these animals were allowed to deliver their pups naturally.

(2) Acupoints were selected according to Zhenqiu and Yongyong [2]. Electroacupuncture was applied as delivery was induced.

In S, the Sanyinjiao (SP 6) group, needles were inserted into a point 10 mm above the medial malleolus of the two hindlimbs, with depth of 3 mm deep, and another needle was inserted 1 mm adjacent into the same point.

In H, the Hegu (LI 4) group, needles were inserted at a point between the first and second phalanx of the two forelimbs, with depth of 1 mm, and another needle was inserted 1 mm adjacent into the same point.

H&S: the above methods were applied in the Hegu (LI 4) and Sanyinjiao (SP 6) group.

In X, the Xuehai (SP 10) group, needles were inserted on the medial aspect of the thigh, the point of lower 1/9 on the line between mediosuperior border of the patella and pubic symphysis, with a depth of 5 mm and another needle was inserted 1 mm adjacent into the same point.

To avoid short circuit, the two needles should not be connected. Needles were stabilized in place with adhesive tape and connected with electroacupuncture therapeutic instrument, with the same pair of positive and negative electrodes at the same point. Slight tremble of limbs was considered to be Deqi sensation [3].

The parameters of electroacupuncture were as follows: 2/100 Hz frequency with automatic shifting between 2 and 100 Hz, 9 V voltage, 0.1 to 0.3 mA intensity, 0.2~0.6 mS pulse width. Each stimulation cycle lasted 20 min, and stimulation was given every 2 hours until the last rat fetus was born. Needles were electrically stimulated with HANS electroacupuncture therapeutic instrument (LH202H, Beijing Huawei Co., Ltd., Beijing, China), and needles were 0.17 mm thick × 7 mm long (Tianjin Xinglin College Medical Instrument Co., Ltd., Tianjin, China).

(3) Medication (MP) group: after delivery started, meperidine was administrated by subcutaneous injection using a dose calculated by a body surface area conversion factor [4]. Meperidine was produced by Qinghai Pharmaceutical Factory Co., Ltd. (Xining, China, License: H63020022).

### 2.3. Nociceptive Testing [5]

The warm water tail-flick test was used to determine pain threshold. Test was conducted before and after treatment and was stopped shortly after the delivery of the first pup. 4 cm of the rat tail was placed in 50 ± 0.5°C warm water and the time between tail input and withdrawal from the water was recorded (3 tests were conducted and the average in units of seconds was recorded). Because pain threshold may appear at different times after acupuncture, the test was conducted 5 times, before acupuncture and at 10, 20, 30, and 60 min after acupuncture, and the largest values were used for statistical analysis. All data are expressed as mean ± SD. STATA 20.0 software package was used for analysis and paired-sample *t*-test was used for comparisons of before and after acupuncture. One-way ANOVA was used for comparisons between the groups.

### 2.4. Specimen Collection

After treatment and delivery, rats were anesthetized with 10% chloral hydrate by abdominal injection at a dose of 400 mg/kg and then decapitated and cleaned with saline. Thereafter, samples were taken from the cerebral cortex and the enlarged segment of spinal cord and kept at −80°C for real-time PCR and western blotting. All samples were tested within 3 months.

### 2.5. Experimental Procedures

#### 2.5.1. Serum NE

Rat NE ELISA kits (TSZ, UAS, FA02097B) were purchased from Shanghai Kexing Trade Company and were used according to the manufacturer's instructions.

#### 2.5.2. Real-Time PCR for Analysis of NET mRNA Expression in the Central Nervous System

The Trizol method was used to extract RNA from the cerebral cortex and enlarged segment of spinal cord, and RNA was reverse-transcribed to cDNA and then kept in −20°C for use. ABI Step One fluorescence reaction PCR instrument was used to measure gene expression. Primer sequences were generated with Primer 5.0 system on NCBI website and designed and synthesized by Shanghai Sangon Biotechnology Ltd.: housekeeping gene (*β*-actin) primers: F: 5′-CGTAAAGACCTCTATGCCAACA-3′ and R: 5′-CGGACTCATCGTACTCCTGCT-3′ amplified with a product of 229 bp. NET primers F: 5′ GAGCTTTGTTATTACTTCATGTCCC 3′ and R: 5′TGCCTTCTCAATGCTACCCA 3′ amplified with a product of 136 bp and *α*2AR primers F: 5′-ACACTCGAGGGATCCTGGCCT,CTCTCGGATC-3′ and Rn: 5′ACAA AGCTTGGGCGCAAAGCTGCCCTCGG-3′ amplified with a product of 217 bp. The amplification conditions were 95°C for 2 min, 95°C for 10 s, and extension step at 60°C for 40 s, for 40 cycles. Relative expression was calculated = 2^−ΔCt^, ΔCt = Ct (target gene) − Ct (housekeeping gene).

#### 2.5.3. Western Blot Analysis to Test Expression of NE and *α*2AR in the Central Nervous System

Total protein extract was extracted from the cerebral cortex and the enlarged segment of lumbar spinal cord, and 120 mg was weighed. 500 *μ*L of precooled cell lysis buffer was added for pyrolysis at 4°C for 30 min followed by centrifugation (4°C at 12000 rpm for 20 min) and protein extraction. Sample loading buffer was diluted into the samples, boiled for 4 min, and kept in −80°C for later use. Protein was separated by SDS-PAGE until the bromophenol blue indicatrix reached the edge of the gel. The protein was transferred to a polyvinylidene difluoride membrane (250 mA, 90 min) which was removed and blocked with 5% nonfat milk by shaking for 1-2 h at room temperature. Primary antibody was incubated with the blocked membrane and then kept at 4°C overnight. On the next day, protein was removed by washing with PBS (15 min × 1 time), and the membrane was washed with TBST 4 times (5 min each time), and incubated with secondary antibody at room temperature for 1 h. After washing with TBST, color developer was added to the front of the PVDF membrane and four-star image analysis system was used to analyze the intensity of the target protein. Biovision BCA Protein Assay Kit, Santa Cruz NET, *α*2AR and *β*-actin primary antibodies, and rabbit secondary antibody, and the chemiluminescence kit were all used according to manufacturer's instructions.

### 2.6. Statistical Analysis

All statistical analyses were performed with SPSS 20.0 for Windows. All data are presented as mean ± standard deviation ($\overset{-}{x}\pm s$). One-way ANOVA was performed for comparisons between multiple groups. The least significant difference was used for comparison of means between groups. A value of *P* < 0.05 was considered as statistically significant.

## 3. Results

### 3.1. Comparison of Pain Threshold between the 6 Groups

**Figure 1:**
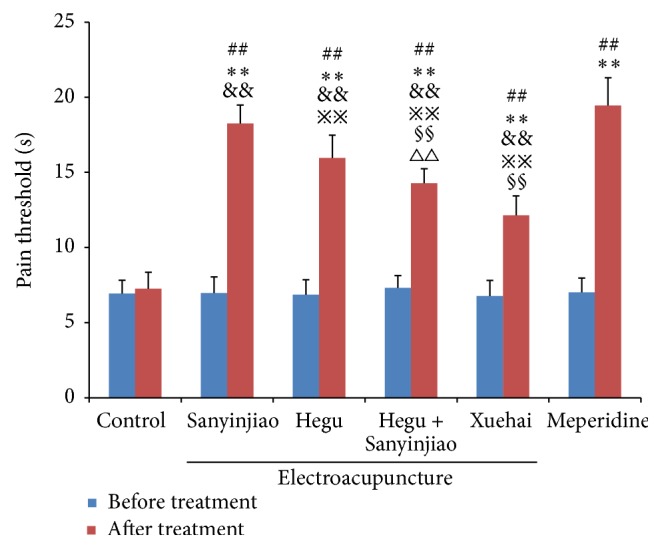
Effects of electroacupuncture on pain threshold of laboring rats. Before and after treatment with electroacupuncture, pain in all rats undergoing parturition was evaluated by the warm water tail-flick test. ^##^ *P* < 0.01 versus before treatment; ^*∗∗*^ *P* < 0.01 versus control; ^&&^ *P* < 0.01 versus meperidine; ^§§^ *P* < 0.01 versus Hegu; ^*※※*^ *P* < 0.01 versus Hegu + Sanyinjiao; ^∆∆^ *P* < 0.01 versus Xuehai. *N* = 20 for each group.

There was no difference in pain threshold in the 6 groups before treatment (all *P* > 0.05), and significant increases were seen after treatments (all *P* < 0.01). LSD multiple comparisons further showed that pain threshold was in the order of control group < X < H&S < H < S < MP group (Figure 1 and Table 1).

### 3.2. Serum NE

**Figure 2:**
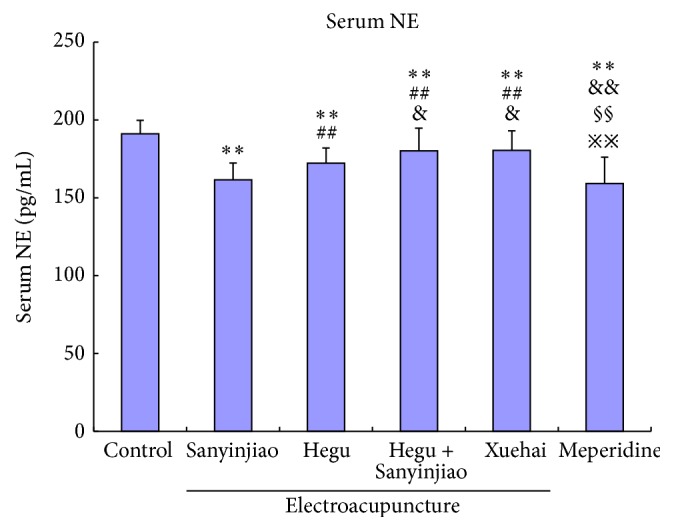
Comparison of serum NE: with control group ^*∗∗*^ *P* < 0.01; with Sanyinjiao (SP 6) group, ^##^ *P* < 0.01; with Hegu (LI 4) group, ^&^ *P* < 0.05, ^&&^ *P* < 0.01; with Hegu (LI 4) and Sanyinjiao (SP 6) group, ^§§^ *P* < 0.01; with Xuehai (SP 10) group, ^*※※*^ *P* < 0.01. *N* = 20 for each group.

After treatment, there were significant decreases in serum NE expression level between the 6 groups in the order of control group < X = H&S = H < S < MP group. Among different groups, the levels in the S group decreased the most (Figure 2 and Table 2).

### 3.3. NET and *α*2AR Protein Expression in the Central Nervous System

**Figure 3:**
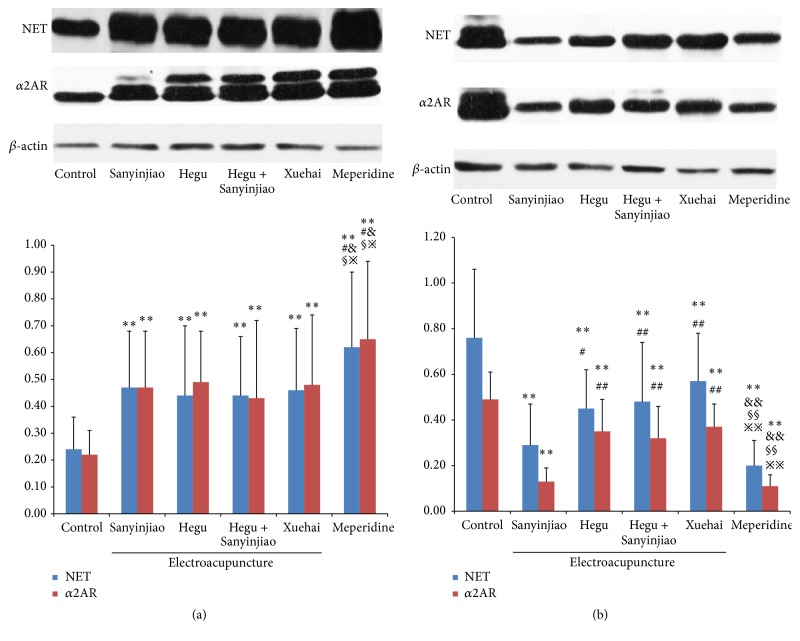
Comparison of NET and *α*2AR protein expression in the central nervous system; (a) gray matter and (b) lumbar spinal cord. The western blots shown are the representative images. With control group ^*∗∗*^ *P* < 0.01; with Sanyinjiao (SP 6) group, ^#^ *P* < 0.05, ^##^ *P* < 0.01; with Hegu (LI 4) group, ^&^ *P* < 0.05, ^&&^ *P* < 0.01; with Hegu (LI 4) and Sanyinjiao (SP 6) group, ^§^ *P* < 0.05, ^§§^ *P* < 0.01; with Xuehai (SP 10) group, ^*※*^ *P* < 0.05  ^*※※*^ *P* < 0.01. *N* = 20 for each group.

Western blot was used to measure NET and *α*2AR protein expression in the central nervous system. Analysis of variance was performed for the cerebral cortex NET (*F* = 5.689, *P* < 0.01) and *α*2AR (*F* = 6.882, *P* < 0.01). There were significant increases between the 6 groups in NET and *α*2AR protein expression (*P* < 0.01) by LSD multiple comparisons in the order of control group < X = H&S = H = S < MP group, with no difference between the electroacupuncture groups. However, there were significant decreases in the enlarged segment of the spinal cord with NET (*F* = 14.171, *P* < 0.01) and *α*2AR (*F* = 35.373, *P* < 0.01) by LSD multiple comparisons in the order of control group < X = H&S = H = S < MP group (Figure 3 and Table 3).

### 3.4. NET and *α*2AR mRNA Expression in the Central Nervous System

**Figure 4:**
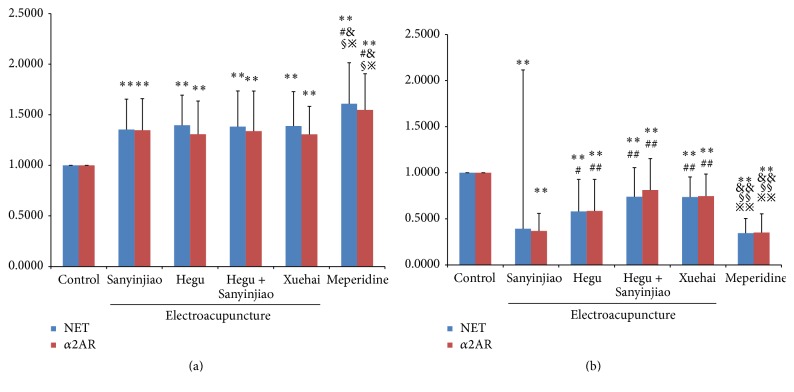
Comparison of NET and *α*2AR mRNA expression in the central nervous system; (a) gray matter and (b) lumbar spinal cord. The western blots shown are the representative images. With control group ^*∗∗*^ *P* < 0.05,  ^*∗∗*^ *P* < 0.01; with Sanyinjiao (SP 6) group, ^#^ *P* < 0.05,  ^##^ *P* < 0.01; with Hegu (LI 4) group, ^&^ *P* < 0.05, ^&&^ *P* < 0.01; with Hegu (LI 4) and Sanyinjiao (SP 6) group, ^§^ *P* < 0.05, ^§§^ *P* < 0.01; with Xuehai (SP 10) group, ^*※*^ *P* < 0.05,  ^*※※*^ *P* < 0.01. *N* = 20 for each group.

Real-time PCR analysis of NET and *α*2AR showed significant differences in the cerebral cortex mRNA expression between the 6 groups (*F* = 7.868, *P* < 0.01) and *α*2AR (*F* = 6.517, *P* < 0.01). Using LSD multiple comparisons NET and *α*2AR mRNA expression increased significantly in all electroacupuncture groups and the MP group in the order of control group < X = H&S = H = S < MP group, with no difference between electroacupuncture groups. Similar changes were seen in mRNA expression in the enlarged segment of the spinal cord for with NET (*F* = 22.543, *P* < 0.01) and *α*2AR (*F* = 21.576, *P* < 0.01; Figure 4 and Table 4).

## 4. Discussion

It is believed in traditional Chinese medicine that labor pain during delivery results from uterine contraction, fetal head descending, and pressure on local tissues and from disharmony of Qi and blood due to fear and nervousness, which is roughly translated as “stagnation results in pain.” It is said in the chapter of* Nine Needles Arid Twelve Yuan* of the* Miraculous Pivot* that needles could be used to unblock meridians and regulate Qi and blood [6]. Thus, it is the belief that acupuncture can be used to relieve pain.

S is the crossing point of the kidney, spleen, and liver meridians. H is the source point of the large intestine meridian of Hand-Yangming and Yangming meridians and is characterized by plenty of Qi and blood. Qi is the master of blood, and blood would follow if Qi moves. H&S is a match with distal and proximal acupoints. X is the point of the spleen meridian. In meridian theory, the uterus is directly connected with the Chong, Ren, and Du meridians, which all correspond to the kidney, spleen, and liver meridians [6]. In this study, electroacupuncture was applied to stimulate different acupoints to activate the Qi of the kidney, spleen, liver, and Ren meridians to unblock meridians and thus regulate Qi and blood and calm mind. In addition, it can stimulate the sensory fibers of nerves in order to relive pain.

In modern medicine, labor pain results from uterine contraction and cervical dilation during the first stage and the fetus descending during the second and third stages of labor. Along with anxiety, fear, and nervousness, sympathetic activation is increased which leads to an increase in NE secretion followed by an increase in pain sensitivity [7]. NE spreads throughout the nervous system and internal organs, and NE neurons mostly exist in the midbrain reticular formation, the locus coeruleus, and the ventrolateral part of the medulla.

NE can act as a hormone in almost every internal organ and it is a neurotransmitter of the sympathetic nerve and central nervous system. It was one of the neurotransmitters that had been the focus of early acupuncture research [8]. It was found that intracerebroventricular injection of NE can antagonize morphine, indicating that NE plays a role in counteracting its analgesic in the brain. When Dawson-Basoa [9] applied electroacupuncture on rats after intraventricular injection of dioctyl phthalate (DOP), he demonstrated that there was an increase in circulating NE and also found a significant decrease in analgesia efficiency from electroacupuncture. On the contrary, intraspinal injection of DOP resulted in an increased efficiency. It was suggested that an increased release of NE in brain counteracts the analgesic effect of electroacupuncture. However, an increasing release of NE in the spinal cord led to strengthening of the effect of electroacupuncture. However, a feedback mechanism whereby NET acted to uptake NE back to the presynaptic membrane occurred. This helped to regulate the NE concentration in the synaptic cleft and to terminate nerve impulse signals and thus to maintain the sensitivity of receptors to the neurotransmitter [10]. In recent years, this has been a research focus as a target for antidepressants and anti-drug-abuse therapy [11].

In the animal neuralgia model, norepinephrine inhibitor experiments showed an analgesia effect, and spinal nerve ligation (SNL) experiments showed an increase of NET, which indicated the analgesic mechanism of transporter-targeted antidepressant [12]. Research suggested that it is essential to inhibit norepinephrine transporter for the synergism of analgesic effect from monoamine transmitter reuptake inhibitors and of opioid drugs [13]. Cerebral NET activity was associated with agitation of PTSD (posttraumatic stress disorder) patients [14]. As a membrane-bound G protein-coupled receptor, *α*2AR can amplify and transduce the extracellular stimulating signal into cells to trigger intracellular biological reactions. It has a feedback inhibitory effect on NE secretion. Research in recent years [15] indicated that there was no visible side effect on respiratory depression and the gastrointestinal system when *α*2AR agonist in the form of opioids was administered for acute pain.

As mentioned above, NET can reuptake NE to the presynaptic membrane so as to regulate the NE concentration in the synaptic cleft [16]. Alpha 2AR also has a feedback inhibitory effect on NE secretion. It has therefore been suggested that NE plays a role in the development of pain by NET and *α*2AR.

In this research, a significant increasing expression of NET, *α*2AR protein, and mRNA in the cerebra and a decrease in the spinal cord were found in electroacupuncture groups. It is speculated that the electroacupuncture stimulation could activate NET and *α*2AR on the presynaptic membrane of the central noradrenergic nerve, resulting in an increased expression of NET and *α*2AR mRNA and protein. This would lead to an increased reuptake of NE by NET, strengthened feedback inhibition of *α*2AR to NE, and thus a decrease of NE release. Moreover, spinal reflex is controlled by cerebra. When cerebral NE release is decreased, the expression of NET and *α*2AR in spinal cord would also reduce, leading to a decreased reuptake of NE by NET, leading to a decreased feedback inhibition of *α*2AR to NE and thus an overall increase of NE release.

As for the different results obtained from different acupoints, this may be related to the neuroanatomical location of the acupoints. The sympathetic nerve controlling the uterus is from T10-S2 [17] and parasympathetic nerve is from L6-S1 [18, 19], which means that the nerve controlling the uterus in the rat is from T10-S2, and the peaks are from T13-L2 and L6-S10. The nerve controlling Sanyinjiao (SP 6) is from L3-6, the nerve controlling Hegu (LI 4) is from C5-T1, and the nerve controlling Xuehai (SP 10) is from L3-4 [20]. This could have led to the different effects being seen on the different acupoints.

This research indicates that the role of NE in the analgesic mechanism of acupuncture is related to reuptake of NE by NET and feedback inhibition of *α*2AR. Under the effect of electroacupuncture, there was an increasing reuptake of NE by NET in the cerebra, strengthened feedback inhibition of *α*2AR to NE, and thus a decrease of NE release. In addition a decreasing reuptake of NE by NET in spinal cord was also seen leading to a decreasing feedback inhibition of *α*2AR to NE and thus an increase of NE release.

## Figures and Tables

**Table 1:** Comparison of pain threshold in the 6 groups ($\bar{x}\pm s$, unit S).

Group	*n*	Before treatment	After treatment
Control	20	6.94 ± 0.89	7.26 ± 1.09
Electroacupuncture Sanyinjiao	20	6.98 ± 1.06	18.26 ± 1.21^##*∗∗*&&^
Electroacupuncture Hegu	20	6.86 ± 1.00	15.96 ± 1.52^##*∗∗*&&*※※*^
Electroacupuncture Hegu + Sanyinjiao	20	7.31 ± 0.82	14.28 ± 0.97^##*∗∗*&&*※※*§§△△^
Electroacupuncture Xuehai	20	6.77 ± 1.04	12.14 ± 1.30^##*∗∗*&&*※※*§§^
Meperidine	20	7.01 ± 0.96	19.45 ± 1.84^##*∗∗*^

*F*		0.736	216.361
*P*		*P* = 0.598 > 0.05	*P* = 0.000 < 0.01

^##^ *P* < 0.01 versus that before treatment; ^*∗∗*^ *P* < 0.01 versus control group; ^&&^ *P* < 0.01 versus meperidine group; ^§§^ *P* < 0.01 versus Hegu (LI 4) group; ^*※※*^ *P* < 0.01 versus Hegu (LI 4) and Sanyinjiao (SP 6) group; ^△△^ *P* < 0.01 versus Xuehai (SP 10) group.

**Table 2:** Comparison of serum NE after treatment ($\bar{x}\pm s$).

Group	*n*	NE (pg/mL)
Control	20	191.17 ± 8.61
Electroacupuncture Sanyinjiao	20	161.54 ± 10.87^*∗∗*^
Electroacupuncture Hegu	20	172.25 ± 9.79^*∗∗*##^
Electroacupuncture Hegu + Sanyinjiao	20	180.23 ± 14.52^*∗∗*##&^
Electroacupuncture Xuehai	20	180.61 ± 12.50^*∗∗*##&^
Meperidine	20	159.18 ± 17.02^*∗∗*&&§§*※※*^

*F*		19.182
*P*		*P* < 0.01

Note: comparison of serum NE: with control group ^*∗∗*^ *P* < 0.01; with Sanyinjiao (SP 6) group, ^##^ *P* < 0.01; with Hegu (LI 4) group, ^&^ *P* < 0.05, ^&&^ *P* < 0.01; with Hegu (LI 4) and Sanyinjiao (SP 6) group, ^§§^ *P* < 0.01; with Xuehai (SP 10), ^*※※*^ *P* < 0.01.

**Table 3:** Comparison of gray value of NET and *α*2AR in the central nervous system of the rats.

Group	*n*	Gray matter region		Lumbar spinal cord	
		NET	*α*2AR	NET	*α*2AR
Control	20	0.24 ± 0.12	0.22 ± 0.09	0.76 ± 0.30	0.49 ± 0.12
Electroacupuncture Sanyinjiao	20	0.47 ± 0.21^*∗∗*^	0.47 ± 0.21^*∗∗*^	0.29 ± 0.18^*∗∗*^	0.13 ± 0.06^*∗∗*^
Electroacupuncture Hegu	20	0.44 ± 0.26^*∗∗*^	0.49 ± 0.19^*∗∗*^	0.45 ± 0.17^*∗∗*#^	0.35 ± 0.14^*∗∗*##^
Electroacupuncture Hegu + Sanyinjiao	20	0.44 ± 0.22^*∗∗*^	0.43 ± 0.29^*∗∗*^	0.48 ± 0.26^*∗∗*##^	0.32 ± 0.14^*∗∗*##^
Electroacupuncture Xuehai	20	0.46 ± 0.23^*∗∗*^	0.48 ± 0.26^*∗∗*^	0.57 ± 0.21^*∗∗*##^	0.37 ± 0.10^*∗∗*##^
Meperidine	20	0.62 ± 0.28^*∗∗*#&§*※*^	0.65 ± 0.29^*∗∗*#&§§*※*^	0.20 ± 0.11^*∗∗*&&§§*※※*^	0.11 ± 0.05^*∗∗*&&§§*※※*^

*F*		5.689	6.88	14.171	35.373
*P*		*P* < 0.01	*P* < 0.01	*P* < 0.01	*P* < 0.01

Note: comparison of NET and *α*2AR mRNA expression in central nervous system, significant difference *P* < 0.05. With control group ^*∗∗*^ *P* < 0.01; with Sanyinjiao (SP 6) group, ^#^ *P* < 0.05, ^##^ *P* < 0.01; with Hegu (LI 4) group, ^&^ *P* < 0.05, ^&&^ *P* < 0.01; with Hegu (LI 4) and Sanyinjiao (SP 6) group, ^§^ *P* < 0.05, ^§§^ *P* < 0.01; with Xuehai (SP 10), ^*※*^ *P* < 0.05, ^*※※*^ *P* < 0.01.

**Table 4:** Comparison of NET and *α*2AR mRNA expression in central nervous system.

Group	*n*	Gray matter region		Lumbar spinal cord	
		NET	*α*2AR	NET	*α*2AR
Control	20	1.0000	1.0000	1.0000	1.0000
Electroacupuncture Sanyinjiao	20	1.3535 ± 0.3008^*∗∗*^	1.3465 ± 0.3124^*∗∗*^	0.3931 ± 1.7217^*∗∗*^	0.3689 ± 0.1891^*∗∗*^
Electroacupuncture Hegu	20	1.3961 ± 0.2977^*∗∗*^	1.3068 ± 0.3293^*∗∗*^	0.5793 ± 0.3482^*∗∗*#^	0.5862 ± 0.3410^*∗∗*##^
Electroacupuncture Hegu+ Sanyinjiao	20	1.3823 ± 0.3527^*∗∗*^	1.3381 ± 0.3963^*∗∗*^	0.7393 ± 0.3158^*∗∗*##&^	0.8124 ± 0.3414^*∗*##&&^
Electroacupuncture Xuehai	20	1.3883 ± 0.3400^*∗∗*^	1.3063 ± 0.2767^*∗∗*^	0.7358 ± 0.2178^*∗∗*##&^	0.7460 ± 0.2394^*∗∗*##&^
Meperidine	20	1.6084 ± 0.4064^*∗∗*#&§*※*^	1.5475 ± 0.3585^*∗∗*#&§*※*^	03446 ± 0.1606^*∗∗*&&§§*※※*^	0.3515 ± 0.203^*∗∗*&&§§*※※*^

*F*		7.868	6.517	22.243	21.576
*P*		*P* < 0.01	*P* < 0.01	*P* < 0.01	*P* < 0.01

Note: comparison of NET and *α*2AR mRNA expression in cerebral cortex and spinal cord, significant difference *P* < 0.05. With control group ^*∗*^ *P* < 0.05, ^*∗∗*^ *P* < 0.01; with Sanyinjiao (SP 6) group, ^#^ *P* < 0.05, ^##^ *P* < 0.01; with Hegu (LI 4) group, ^&^ *P* < 0.05, ^&&^ *P* < 0.01; with Hegu (LI 4) and Sanyinjiao (SP 6) group, ^§^ *P* < 0.05, ^§§^ *P* < 0.01; with Xuehai (SP 10), ^*※*^ *P* < 0.05, ^*※※*^ *P* < 0.01.
